# Repetition without Repetition or Differential Learning of Multiple Techniques in Volleyball?

**DOI:** 10.3390/ijerph181910499

**Published:** 2021-10-06

**Authors:** Julius B. Apidogo, Johannes Burdack, Wolfgang I. Schöllhorn

**Affiliations:** 1 Department of Training and Movement Science, Institute of Sport Science, Johannes Gutenberg-University Mainz, 55099 Mainz, Germany; japidogo@uni-mainz.de (J.B.A.); wolfgang.schoellhorn@uni-mainz.de (W.I.S.); 2 Akanten Appiah-Menka University of Skills Training and Entrepreneurial Development, Kumasi AK-039, Ghana

**Keywords:** motor learning, differential learning, volleyball, overhand service, overhand pass, underhand pass, multiple techniques, skill acquisition

## Abstract

A variety of approaches have been proposed for teaching several volleyball techniques to beginners, ranging from general ball familiarization to model-oriented repetition to highly variable learning. This study compared the effects of acquiring three volleyball techniques in parallel with three approaches. Female secondary school students (N = 42; 15.6 ± 0.54 years) participated in a pretest for three different volleyball techniques (underhand pass, overhand pass, and overhead serve) with an emphasis on accuracy. Based on their results, they were parallelized into three practice protocols, a repetitive learning group (RG), a differential learning group (DG), and a control group (CG). After a period of six weeks with 12 intervention sessions, all participants attended a posttest. An additional retention test after two weeks revealed a statistically significant difference between DG, RG, and CG for all single techniques as well as the combined multiple technique. In each technique—the overhand pass, the underhand pass, the overhand service, and the combination of the three techniques—DG performed best (each *p* < 0.001).

## 1. Introduction

Coaches and physical education teachers are always faced with the challenge of teaching multiple techniques and fostering athlete performance in the most time efficient manner. To achieve this goal, coaches and athletic trainers are always looking for the most effective and efficient learning approaches. The four most popular and widely used approaches to teaching and improving performance and learning, which include innovative elements that had not been previously considered, are listed in a historical order:

(a) The repetition method approach. This method was first mentioned by Plato (450 B.C.) in the context of learning by contrast and was later investigated in more detail by Gentile [1]. The repetitive method approach is based on the assumption that there is an ideal type of movement that can be perfected by several repetitions of the target movement during the learning process. This method is still considered the method of choice by many physical education teachers and coaches.

(b) The original purpose of the methodical series of exercise approach [2] is to learn more complex target movements through a streamlined (blocked) sequence of preliminary exercises increasingly similar to the target movement. In this process, each preparatory exercise follows the logic of the RM. This method is still chosen the most for learning singular complex movements.

(c) The variability of practice approach [3] is based on Schmidt’s schema theory [4], which coarsely indicates that invariant elements such as relative timing or relative forces of an already automatized movement become more stable when trained in combination with variable parameters such as absolute forces or absolute durations. Nevertheless, each exercise is trained repetitively (=blocked), oriented on subgoals as prototype. Although the area of application was limited to movements without the influence of gravitational forces [5], this approach inspired teachers and coaches to make the training of a single technique more variable once it has been learned.

(d) The contextual interference was originally operationalized by Battig [6,7] in the context of verbal learning and later applied to fine motor learning by Shea and Morgan [8]. From its origin, contextual interference approach is a learning approach in which one skill is practiced in the context of other skills. The approach is typically associated with two phenomena, namely impaired acquisition on the one hand, and enhanced learning on the other [9,10]. Three models from cognitive psychology have been proposed to explain these phenomena: the elaboration [11], the reconstruction [12], and the retroactive inhibition hypothesis [13]. All three models assume that a higher cognitive effort is required for the random schedule compared to a blocked schedule, which is typically associated with immediately poorer performance due to working memory overload but leads to better retention.

Originally focused only on the learning of a single (text) motion interspersed with additional motions (=context), the contextual interference approach is now primarily investigated for the parallel learning of multiple movements. The approach is still struggling with its application in sports practice, since, among other things, systematic effects have only been found for movements with a small number of degrees of freedom [10,14] despite isolated evidence of positive effects in movements with more degrees of freedom [15].

(e) The differential learning approach [16,17] assumes that improving the performance or learning of a movement depends largely on an individual’s characteristics and experiences, which are assumed to be embodied in individual neuro-(muscular) structures that need to be stimulated individually in varying contexts in order to achieve an effective restructuring for changes in behavior. The reciprocal matching of the exercises provided by the trainer to the nature or extent of the learner’s individual variations is described by the principle of stochastic resonance [18,19,20]. Because the differential learning approach is the most recent approach proposed to increase technical performance and since it is the primary subject of the study, it is discussed in some detail below.

The parallel observation of analogies related to fluctuations in three research areas served as the inspiration for the differential learning approach. First, within the research on the identification of individual movement patterns, constant fluctuations of biomechanical parameters were observed [21,22,23]; second, fluctuations in the field of dissipative dynamic systems were assigned an essential role especially in phase transitions [24]; third, in the field of research on artificial neural networks, it was known that they perform better when added with noisy information during the training phase [25,26,27]. It is postulated that the learner’s behavior should be the focus of interest rather than the idea of a collective movement ideal. Supposedly destructive deviations from the movement ideal became reinterpreted as constructive fluctuations that should make the learning system unstable and enable self-organized learning [17,19]. Whereas differential learning was initially applied and studied only in sports for learning and improving individual techniques [18,28,29,30], there are now also confirming studies on its effectiveness in fine motor [31,32,33] or everyday movements, as well as in the field of tactics [34,35], strength [36,37], and endurance training. Isolated studies on the parallel acquisition of two techniques [38,39] suggest its application in the learning of multiple techniques as well.

The only approach that so far tries to explain the learning of multiple techniques is the contextual interference approach. However, the studies on the simultaneous acquisition of multiple techniques in volleyball using this approach have led to ambiguous results. The acquisition of two volleyball techniques showed partial or no support for benefits of contextual interference in the form of random compared to blocked order [40]. Fialho, Benda, and Ugrinovich [41] could not find significant differences between blocked and interleaved training groups for either the post or the retention test when training two service techniques. Similar results were provided by a study on training two techniques (overhand and underhand service) [42] under the contextual interference approach. Several other studies [43,44,45] also failed to find significant effects of training condition in acquisition or retention performance when training the three basic volleyball techniques. In contrast, when training the same three techniques, Bortoli et al. [46] reported better transfer for the random and serial practice groups than for the blocked group.

Interestingly, all of these studies were conducted on adolescent participants with an average age between 12.4 years [45] and 16.3 years [41]. None of these studies could fully substantiate the two contextual interference related phenomena; only one study [46] partially verified the advantageous learning effect. In contrast, a study of three volleyball techniques with adult students with an average age of 21.5 years found verification of the full contextual interference effect with impaired acquisition and increased retention [47]. Taken together, all these studies on volleyball suggest that the contextual interference approach should be restricted to adults [48]. Whereas most contextual interference studies on athletic movements investigated the parallel training of similar techniques within a sport and found largely consistent changes for this, the parallel acquisition of a running, a jumping, and a throwing movement showed discipline-specific trajectories during the learning process [46].

Apparently, the contextual interference approach does not provide a model that can explain the different results comprehensively. As suggested above, the differential learning approach may provide a more general and appropriate framework for understanding movement learning, at least in movements with more degrees of freedom. In order to increase external validity by further approximating practical, realistic learning, this study aims to investigate the effect of differential learning training on the parallel acquisition and learning of the three volleyball techniques mainly used and taught by beginners. The expectation, based on previous studies, is that students taught using the differential learning approach will increase their performance on the posttest and retention test more than students using the repetitive training method or the general ball familiarization. To what extent an extension of the applications of the differential learning approach for novices can be recommended and to what extent the performance developments of the three approaches differ are the questions that will be investigated. 

## 2. Material and Methods

### 2.1. Participants

A total of 42 female volleyball novices (15.6 ± 0.54 years) from several Ghanaian state high schools in Kumasi voluntarily participated in this study. After being informed of the content and purpose of the study, the participants’ parents provided written informed consent. All procedures were conducted according to the guidelines of the Declaration of Helsinki and approved by the Institutional Review Board of Akanten Appiah-Menka University of Skills Training and Entrepreneurial Development (AAMUSTED/K/RO/L.1/219, 31 August 2021).

A pretest in all three techniques was conducted with them in blocked sequence, and the individual scores for each technique were summed up to form individual total scores for each of the participants. Based on the individual results, they were parallelized into three groups of 14 participants each: a repetitive learning group (RG), a differential learning group (DG), and a control group (CG). The individual scores were summed up to represent the group score (group means).

### 2.2. Design

A pre-posttest design with additional retention test (see Section 2.2.2) was chosen for this investigation. The pretest was followed by an intervention period of six weeks with a subsequent posttest and a retention test after another two weeks without intervention. A standardized warm-up was performed before all tests. During the intervention phase, the participants trained twice a week (always on Mondays and Thursdays), where each training session lasted one hour.

#### 2.2.1. Intervention

The RG trained according to the Federation International de volleyball Coaches manual [49]. Each training session was preceded by a five-minute warm-up activity, which consisted of minor games, such as “three-on-one”, “chase and catch”, and “seven-on-one’’. After completing the warm-up activities, the participants proceeded directly to practice in their respective groups. After the group training, the session was finished. The RG training was characterized by taking one of the techniques per session repeating it 15 times in the blocked form, from overhand service (S) to overhand pass (O), to underhand pass (U) (SSS…, OOO…, UUU…) following that order repeatedly with corrective feedback per session.

The DG training corresponded to the training sequence of the RG in block; however, their training was characterized first by no repetitions by adding stochastic perturbations to the three techniques to be learned and second by no corrections. Appendix A Table A1 contains a list of all given tasks from which a number was randomly selected to instruct the group. Each participant in both intervention groups had 15 trials per training session for each technique. In total, each participant had 180 relevant ball contacts over the entire period.

The control group (CG) engaged in ball familiarization games that were not directly related to volleyball, such as ball throwing and catching games.

#### 2.2.2. Test Design

**Figure 1:**
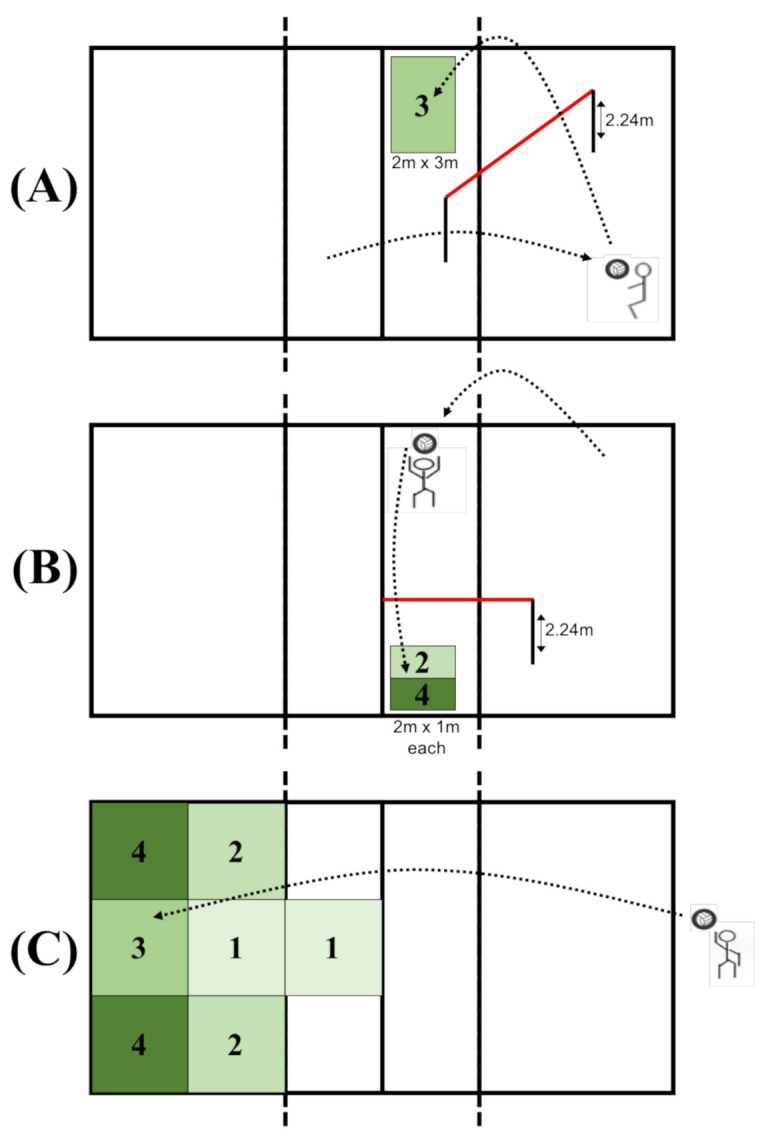
Test designs for the three volleyball techniques including scores. Each subtest corresponds to one technique: (**A**) underhand pass, (**B**) overhead pass, and (**C**) overhead service.

The test as presented in Figure 1 comprised of three subtests, each corresponding to one of the techniques to be learned: underhand pass, overhand pass, overhand service. All subtests were carried out according to the AAHPERD volleyball skill test manual [50] on a regular outdoor volleyball court.

Subtest underhand pass (Figure 1A): To test the underhand pass accuracy, the student stood in a 2 m^2^ square on the right-hand side of the volleyball court (zone Z5) and received a ball thrown from zone 2 of the other court and passed the ball over a rope (height 2.24 m) into a 3 m × 2 m target area in zone Z2 of the participant’s court for which 4 points are awarded if ball lands in the target area and 2 points if it lands on the lines of the target area.

Subtest overhand pass (Figure 1B): To test the accuracy of the overhand pass, the participant stood in zone Z2, received the ball from zone Z6, and passed the ball over a 2.24 m high rope into two 1 m × 2 m target areas, with the one farther from the participant scoring 4 points, the one closer scoring 2 points, and the line in between scoring 3 points.

Subtest overhand service (Figure 1C): The participants stood at the end of the field in a central 2 m wide area and served the ball over their head to the other field over the 2.24 m high net into the 2 m × 2 m rectangular target areas, with points awarded for each area. The further back and sideways the target area that was hit, the more points a serve resulted in, ranging from 1 to 4 points. In between the zones, the two zone points were added and divided by two and the points given.

For all subtests, a score of zero was given if the ball did not land within the target zones or did not touch any of the lines of the marked target areas. The participants performed 10 trials in each subtest. The maximum score for each subtest was 40 points and a minimum of 0. The test was performed in the order from underhand pass to overhand pass to overhand service and on the same day under comparable conditions.

Six research assistants were trained to assist in the process of training and conducting the test. The execution of an attempt was counted only if the ball thrown by the research assistant was receivable by the participant within the marked area. Otherwise, the attempt was repeated.

### 2.3. Data Analysis

The groups were compared statistically based on their results in each technique and in combined multiple techniques. To determine the combined multiple techniques, the mean values of the three individual techniques (overhand pass, underhand pass, and overhand service) were adjusted using z-standardization. To check the internal consistency of the tests for the respective techniques, 10 participants each performed the respective test at intervals of one week. Cronbach’s alpha was determined based on the values from weeks 1 and 2.

Analyses of the data using Shapiro–Wilk tests revealed that some variables violated the assumption of normal distribution. Consequently, the development of the groups across the measurement time points and the comparison of the groups at the respective measurement time points were performed using non-parametric statistical tests.

For the analysis of the development within the groups in the respective techniques at pre-, post-, and retention-test, the results of the tests were statistically compared using Friedman ANOVA. In case of significant results, pairwise Bonferroni-corrected post hoc Dunn–Bonferroni tests were performed.

In order to compare the different groups at the respective pre-, post-, and retention test, the test results of the specific techniques were compared statistically using Kruskal–Wallis tests. The comparison at the time of the pretest here also represents the basis of the test for homogeneity. Significant results were further statistically compared using pairwise Bonferroni-corrected post hoc Dunn–Bonferroni tests. 

In addition, the effect size r was calculated for the pairwise post-hoc tests of the Friedman and Kruskal–Wallis tests, respectively. Thereby, 0.1 ≤ r < 0.3 corresponds to a weak effect, 0.3 ≤ r < 0.5 to a medium effect, and r ≥ 0.5 to a strong effect [51].

The *p*-value at which it is considered worthwhile to continue research [52] was set at *p* = 0.05, with decreasing p increasing the probability that the null hypothesis does not explain all the facts.

## 3. Results

**Figure 2:**
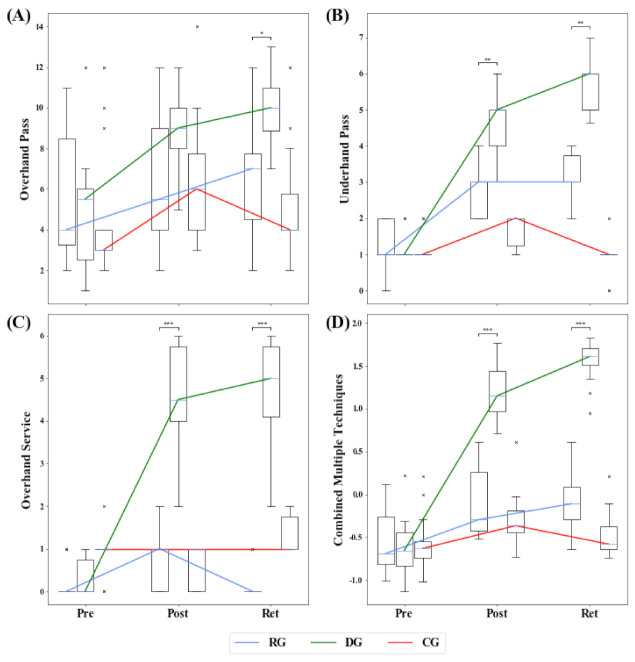
Development of the groups in the test on the respective techniques over the duration of the examination. Values are considered as outliers if they are outside the interval [Q1 − 1.5 * (Q3 − Q1), Q3 + 1.5 * (Q3 − Q1)]. × = outlier (each × stands for one outlier). Brackets show significant differences between RG and DG only. (* *p* ≤ 0.05; ** *p* ≤ 0.01; *** *p* ≤ 0.001). Shown are the boxplots of the overhand pass (**A**), underhand pass (**B**), overhand service (**C**), and combined multiple techniques (**D**). For the clarity of the development of the groups, the median curves are also shown by line plots. RG = repetitive learning group; DG = differential learning group; CG = control group; Pre = pretest; Post = posttest; Ret = retention test.

The proof for internal consistency of the tests showed acceptable or good results for the overhand pass (α = 0.774), underhand pass (α = 0.812), and for combined multiple techniques (α = 0.889) tests. Only the overhand service test was just below the threshold in the questionable interval (α = 0.678). The test results of each technique and the combined z-standardized values of each test are shown in Figure 2A–D. The results of the statistical analyses are presented in Table 1.

### 3.1. Development within Groups over Measurement Time Points

The DG improved statistically significantly over the course of the study in all three techniques and also in the combined multiple techniques (*p* < 0.001) and the effect size was at least medium each time (r > 0.442). In the subtests for the underhand pass, the overhand service, and the combined multiple techniques, there was a statistically significant improvement with a medium effect size in each case in the acquisition phase (*p* < 0.007, r > 0.305) and a further, however, not significant, improvement in the retention phase. Solely in the case of the overhand pass there was only a statistical trend in the acquisition phase (*p* = 0.056, r = 0.256), although there was a significant increase in the retention phase (*p* = 0.024, r = 0.288).

In the overall course, the performance level of RG tended to develop similarly to the DG in the techniques of the underhand pass and in the combined multiple technique. The results showed a significant improvement with a medium effect size (*p* < 0.001, r > 0.391). In both techniques, significant improvement was also observed in the acquisition phase (*p* < 0.004, r > 0.324). However, no significant improvement was shown in the overhand service and the overhand pass.

The performance level of CG never changed significantly, neither positively nor negatively, over the course of the study. Nonetheless, significant improvements from pretest to posttest were seen in the underhand pass and the overhand pass, each followed by significant decreases to the retention test. In the case of the overhand service, the exact opposite development was observed.

### 3.2. Comparison between Groups across Measurement Time Points

An examination of the homogeneity of the three groups on the pre-test using the Kruskal–Wallis test revealed no statistically significant differences (*p* ≥ 0.42) for the overhand and underhand pass as well as for the combined techniques. Only for the overhand service the groups differed significantly (*p* < 0.001), and pairwise Bonferroni-corrected post hoc comparisons revealed significant significantly larger values of the control group to the RG (*p* = 0.002, r = 0.649) and DG (*p* = 0.006, r = 0.580); there were no differences between RG and DG (*p* = 1.000).

Statistically significant global differences were found between groups for the overhand pass, underhand pass, overhand service, and combined multiple techniques in both the posttest and retention test (*p* < 0.021).

For the overhand pass technique, the posttest showed a significant difference with a medium effect size between the DG and the CG (*p* = 0.042, r = 0.465), whereas for the retention test, the DG performed significantly better than the RG (*p* = 0.013, r = 0.550) and the CG (*p* < 0.001, r = 0.732) with each a strong effect size.

Pairwise post hoc comparisons in the underhand pass technique showed that in the posttest and retention test, the DG performed significantly better and with strong effect size than the RG (*p* = 0.005, r = 0.597) and the CG (*p* < 0.001, r = 1.165), with the RG also performing significantly with a medium effect size better than the CG (*p* = 0.050, r = 0.452).

The post hoc tests for the overhand service, although the CG was still significantly better than the RG and DG at pretest, showed that the DG performed significantly better than the RG (*p* < 0.001, r > 0.892) and CG (*p* < 0.006, r > 0.597) in both the post and retention tests with a strong effect size. The RG also scored significantly better with a strong effect size than the CG at the retention test (*p* = 0.012, r = 0.546).

The same picture of DG outperforming RG (*p* < 0.001, r > 0.700) and CG (*p* < 0.001, r > 0.938) in both the post and retention test with strong effect sizes can also be seen in the combined multiple techniques; there was no statistical difference between RG and CG.

## 4. Discussion

The purpose of this study was to compare the effects of the repetition-oriented learning (RG) approach with the differential learning (DG) approach of teaching three volleyball techniques (underhand pass, overhand pass, and overhand service) to adolescent female novices in parallel, compared to general ball familiarization exercises (CG). All three groups started from the same performance level but developed differently depending on the learning approach. The changes of the CG, whose activities can be understood as having no direct relation to volleyball techniques, are statistically within the chance level and represent a fair reference to the other two interventions. The performance of the RG and DG each improved from pre to posttest, with the DG performing better than the RG on average for all comparisons. From the post to the retention test, the DG improved a further time in each technique, although only statistically significantly in the overhand pass. At the time of the retention test, the DG outperformed the RG in every single technique as well as in the multiple technique in a statistically significant manner.

First, we consider the results in the acquisition phase from pretest to posttest. With respect to each of the techniques individually, these findings are in accordance with earlier studies on the comparison of differential learning with repetitive-corrective learning [18,53,54,55,56]. Looking at the results from pre- to posttest as a whole, however, it is somewhat surprising how clearly the DG outperformed the RG in the individual techniques as well as in the combined multiple technique in the posttest and later in the retention test. This suggests that the parallel learning of multiple techniques in one sport with the differential learning approach might have had a particularly positive effect on learning the respective single techniques.

In order to understand the increased learning outcomes of the combination of individual techniques in a theoretical framework, it seems reasonable to use the contextual interference approach, which is currently the most widely used explanatory approach. Interestingly, however, the results contradict explanatory models within the contextual interference theory. These models predicted posttest advantages for the repetition-oriented groups, because the working memories were so adversely overloaded in the DG that it was even more likely to perform worse. The effect had to be larger, because the DG was exposed to such a large number of different movements, which has never been observed in studies on the contextual interference effect. Surprisingly, the absence of posttest benefits on the side of blocked-training groups can also be observed in several contextual interference studies on athletic movements [43,46]. Within the contextual interference framework, the favorable results of the DG group at posttest are even more surprising from another point of view. The theory postulates at least a certain number of correct repetitions of the prototypical movement as a mandatory prerequisite for successful acquisition and learning [11].

In contrast, however, the results of the combined techniques can also be explained by means of the theory of differential learning. According to this approach, training is associated with successful learning even without having performed the “ideal” prototypical movement once, just by practicing mainly movements surrounding the theoretically presumed target movement. According to differential learning theory, a neural network becomes more robust to perturbations, which can be considered as the deviations from a given ideal, when trained with variable input. According to the knowledge of the behavior of artificial neural networks, which derive their original principles from the properties of the neurons [25,26,57,58], differential learning theory expects the system to be trained with additional noise so that the individual not only finds a more global solution [19], but also prepares the system for more and larger deviations from a mentally constructed prototype that are highly likely to occur in the future [59]. Moreover, this noise could be amplified by learning multiple techniques in parallel to the correct degree, which in turn could provide a rationale for more efficient learning. By training an athlete’s neural network for a wider solution space, the system uses the ability to interpolate, which appears to be superior to extrapolation [17]. In addition to what is now generally known about this property in artificial neural networks, Catalano and Kleiner [60] provided evidence for its validity in human behavior. They showed that in speed-based tasks variable-trained participants performed better than block-trained ones not only within the trained range, but also outside of it. Analogously, differential learning theory recommends increasing the range of experience to have a higher probability of using the interpolation in the next movement, which is sure to contain something new. In this context, it is important to mention that the space of solution is an abstract image that is highly dimensional and spanned by all joint angles, angular velocities, angular accelerations, spatial limb orientation, muscles activations, and many other influencing variables. Due to the high dimensionality of the solution space, it seems very unlikely to find variables that do not change individually and situationally. A very first computational approach was suggested with the Uncontrolled Manifold Theory [61], which could so far only be successfully applied to fine motor movements due to the limited number of influencing variables.

Nevertheless, the contextual interference theory has been developed for explaining the interference phenomenon after acquisition that can be systematically observed in fine-motor movements in the laboratory, such as keypress or barrier knock-down tasks. Since differential learning theory has failed to explain this so far, it is strongly recommended to develop differentiated explanatory models for fine and for gross motor movements and abstain from attempts to generalize the theory beyond the original model purpose [62,63]. One starting point could be the differentiated inclusion of various relative demands on different sensory systems during the specific movement task. Whereas the majority of contextual interference experiments for small motor movements contain dominant visual-spatial components (sequence of buttons, sequence of wooden blocks to be thrown) that are primarily processed sequentially, the majority of experiments for large motor sports movements contain a dominant proprioceptive and kinesthetic component that is primarily processed in parallel. When the importance of the visual component increases in gross-motor movements, such as in baseball [64], where the batter’s decision is highly dependent on the ability to read approaching balls with different speeds, spin, and directions, or in basketball [65] and pistol shooting [66], with the problem of estimating the distance to the basket or the launch angle, the likelihood of finding support for the contextual interference paradigm also seems to increase. Whether the relative importance of visual and kinesthetic influence shifts over the course of a long-term learning process or is also age-dependent in volleyball needs to be clarified by future studies. A suggestion to explore the interdependencies of the visual and motor systems is given by Gestalt psychologists with their analysis of the “motor outline of optical shapes” [67].

The relative retention test performances of the RG and the DG can be explicated by both theories, the differential learning and the contextual interference theory. The group with more variation during acquisition performs significantly better than the other. The contextual interference theory, however, only expects better results in the relative comparison, which would occur if both groups decreased in performance, the RG more than the DG. In contrast, based on the majority of experiments to date, differential learning is expected to show a further increase in performance on the retention test compared to the posttest.

If, in addition to the results of the two experimental groups RG and DG, we take into account the results of the control group CG for analysis, we can notice a relation to both the “specificity–versus–generalization” problem [68,69] and the resonance problem [18,19,70], which, strictly speaking, can be considered as the same problem on different scales. Whereas the specificity–versus–generalization problem is binary coded (specific/general), the stochastic resonance with its optimum function is at first sight ternary coded (too much/exact/too little). Because of its continuous frequency and learning rate scale, the SR model can actually be viewed as a decimal scale that provides information on how much noise needs to be added or reduced to achieve optimal resonance. Looking at the higher improvement rates of the two volleyball-specific approaches (RG and DG) after acquisition, it is obvious that the interventions of the CG for volleyball were too general and most likely outside the specific solution space. The interpretation based on the stochastic resonance phenomenon takes it one step further. The previous distinction becomes more sophisticated and, analogous to the other two, identifies too much noise for the CG, but too little noise for the RG and in between the optimal noise in the interventions for the DG.

From a more application-oriented point of view, this study presents a picture that alleged errors in movement during the acquisition process should not be regarded as detrimental to learning, but rather as an advantage for learning [16], indicative of the fact that the learning base has been broadened. This is because in the differential learning paradigm, error identification and correction during acquisition were completely absent, which allowed for self-organization. In the same context, a few issues in motor learning need more attention in terms of classical learning theories:

(1) Traditional motor learning theories point out errors and seek to correct them (motor learning), implying that there is a specific way to perform a movement, technique, or skill [1,11]. These error corrections aim to improve or perfect performance within a feedback loop [71]. However, from the results of the study, it appears that these corrections limit the potential of learners by making them stick to certain movement patterns that are considered the best movements in the techniques to be learned. This in a way controls the actual potential of the learner and leads to role model learning that does not allow the learner’s originality and innovation to shine.
(2) Learning theories also attempt to repeat the movement being learned multiple times in order to perfect the movement. Considering judgment errors only as fluctuations has the potential to destabilize the system to allow true self-organization [16,17,72]. An intermediate step between teacher-oriented and self-organized learning is already offered by reform pedagogical approaches according to Basedow, Pestalozzi, or Dewey, where discovery-based learning is allowed within given limits. Either way, coaches and trainers may have to reconsider what errors are and to rethink the idea of role modeling (ideal movement), because learners are not given the freedom and opportunity to learn and develop naturally as individuals. Again, the physical education teacher’s task to teach gross-motor movements in sports to beginners may involve revising their approach towards their lessons, as well as the methods they employ.

The individual technique performance by the three groups shows that the DG had more participants who improved their performance throughout the experiment than the RG and CG. This is at least an indication that constant repetition—even without “repetition” in Bernstein’s sense [73,74]—is not the only approach to motor skill acquisition, does not really improve performance quickly, can limit the learner, and hardly allows for system adaptation.

This study also had some attendant challenges; (1) the age of the participants, who were female, indicated that they were undergoing physical maturation and development, so they were reluctant to be active during the exercises and tests at a time when some of them had physical changes. (2) Facilities such as playing courts and volleyballs were not enough; at least three playing courts would be better. (3) The single-hour physical education class might have been too short to practice adequately.

## 5. Conclusions

This study compared the effects of the general (CG), repetitive (RG), and differential motor learning (DG) approaches in parallel acquisition of three volleyball techniques (underhand pass, overhand pass, and overhand service). The results indicate the advantages of the differential learning approach in comparison to the general ball familiarization and the repetitive prescriptive approaches not only during the acquisition, but also during the learning phase. Through differential learning, where no movement was repeated, the adolescent girls who were absolute beginners in volleyball seemed to have experienced a broader spectrum of movements compared to before, which allowed their neuro-motor system to adapt more efficiently to the demands of the three techniques.

Previous models of variable [4] or interleaved practice [8] fail to explain this phenomenon, since they assume either memorization of the to-be learned movements or at least parts (invariants) of the movements through correct repetitions. Noisy training is associated with the theory and behavior of artificial neural networks in connection with the principles of system dynamics. In the first case, noise leads to more stability in the subsequent application, and in the second case, the increase of noise is a condition for a self-organized change of states. Although more research will be necessary to understand in detail the form and extent of the variations (noise) on the situations (e.g., learning phases) and individuals or groups, the results in conjunction with the findings of other studies indicate an essential role of increased noise in learning processes of multiple movements. Learning multiple movements in parallel appears to further positively influence this “differential learning effect”. In addition, the highly variable differential learning exercise protocol could expand the space of experience and thus better prepare athletes to solve future problems more adequately, as indicated by the retention tests.

Although the statistics applied do not allow for generalization, the numerous significant results with corresponding effect sizes may, on the one hand, encourage researchers (according to Fisher’s original interpretation of his statistics) to continue the study of differential learning and, on the other hand, provide coaches and physical education teachers with an effective and time-saving method to support motor learning.

## Figures and Tables

**Table 1:** Statistical comparisons at the three measurement time points within and between groups.

Comparison	Friedman-Test or Kruskal-Wallis-Test (Rank Scores)	Post Hoc Dunn-Bonferroni-Tests
Overhand Pass		
RG: Pre—Post—Ret	χ^2^(2) = 3.720, *p* = 0.156 (Pre: 1.61; Post: 2.25; Ret: 2.14)	–
DG: Pre—Post—Ret	χ^2^(2) = 25.529, *p* < 0.001 *** (Pre: 1.04; Post: 1.96; Ret: 3.00)	Pre vs. Ret: *p* < 0.001 ***, r = 0.544 ^+++^ Post vs. Ret: *p* = 0.024 *, r = 0.288 ^+^
CG: Pre—Post—Ret	χ^2^(2) = 11.306, *p* = 0.004 ** (Pre: 1.57; Post: 2.68; Ret: 1.75)	Pre vs. Post: *p* = 0.010 *, r = 0.296 ^+^ Post vs. Ret: *p* = 0.042 *, r = 0.248 ^+^
Pre: RG—DG—CG	χ^2^(2) = 1.709, *p* = 0.426 (RG: 24.14; DG: 22.11; CG: 18.25)	–
Post: RG—DG—CG	χ^2^(2) = 7.758, *p* = 0.021 * (RG: 18.04; DG: 28.89; CG: 17.57)	CG vs. DG: *p* = 0.042 *, r = 0.465 ^++^
Ret: RG—DG—CG	χ^2^(2) = 15.508, *p* < 0.001 *** (RG: 18.36; DG: 31.42; CG: 13.96)	RG vs. DG: *p* = 0.013 *, r = 0.550 ^+++^ DG vs. CG: *p* < 0.001 ***, r = 0.732 ^+++^
Underhand Pass		
RG: Pre—Post—Ret	χ^2^(2) = 21.714, *p* < 0.001 *** (Pre: 1.07; Post: 2.29; Ret: 2.64)	Pre vs. Post: *p* = 0.004 **, r = 0.324 ^++^ Pre vs. Ret: *p* < 0.001 ***, r = 0.420 ^++^
DG: Pre—Post—Ret	χ^2^(2) = 23.306, *p* < 0.001 *** (Pre: 1.00; Post: 2.19; Ret: 2.81)	Pre vs. Post: *p* = 0.007 **, r = 0.319 ^++^ Pre vs. Ret: *p* < 0.001 ***, r = 0.483 ^++^
CG: Pre—Post—Ret	χ^2^(2) = 14.000, *p* < 0.001 *** (Pre: 1.82; Post: 2.64; Ret: 1.54)	Post vs. Ret: *p* = 0.010 *, r = 0.296 ^+^
Pre: RG—DG—CG	χ^2^(2) = 0.392, *p* = 0.822 (RG: 22.79; DG: 20.86; CG: 20.86)	
Post: RG—DG—CG	χ^2^(2) = 31.014, *p* < 0.001 *** (RG: 20.36; DG: 34.50; CG: 9.64)	RG vs. DG: *p* = 0.005 **, r = 0.597 ^+++^ RG vs. CG: *p* = 0.05 *, r = 0.452 ^++^ DG vs. CG: *p* < 0.001 ***, r = 1.049 ^+++^
Ret: RG—DG—CG	χ^2^(2) = 36.687, *p* < 0.001 *** (RG: 21.43; DG: 35.00; CG: 7.57)	RG vs. DG: *p* < 0.008 **, r = 0.577 ^+++^ RG vs. CG: *p* < 0.005 **, r = 0.589 ^+++^ CG vs. DG: *p* < 0.001 ***, r = 1.165 ^+++^
Overhand Service		
RG: Pre—Post—Ret	χ^2^(2) = 8.000, *p* = 0.018 * (Pre: 1.86; Post: 2.43; Ret: 1.71)	–
DG: Pre—Post—Ret	χ^2^(2) = 21.347, *p* < 0.001 *** (Pre: 1.00; Post: 2.35; Ret: 2.65)	Pre vs. Post: *p* = 0.002 **, r = 0.360 ^++^ Pre vs. Ret: *p* < 0.001 ***, r = 0.442 ^++^
CG: Pre—Post—Ret	χ^2^(2) = 9.172, *p* = 0.010 * (Pre: 1.96; Post: 1.61; Ret: 2.43)	–
Pre: RG—DG—CG	χ^2^(2) = 14.235, *p* < 0.001 *** (RG: 16.39; DG: 17.86; CG: 30.25)	RG vs. CG: *p* = 0.002 **, r = 0.649 ^+++^ DG vs. CG: *p* = 0.006 **, r = 0.580 ^+++^
Post: RG—DG—CG	χ^2^(2) = 29.276, *p* < 0.001 *** (RG: 14.39; DG: 35.43; CG: 14.68)	RG vs. DG: *p* < 0.001 ***, r = 0.892 ^+++^ DG vs. CG: *p* < 0.001 ***, r = 0.879 ^+++^
Ret: RG—DG—CG	χ^2^(2) = 35.229, *p* < 0.001 *** (RG: 8.21; DG: 34.85; CG: 20.93)	RG vs. DG: *p* < 0.001 ***, r = 1.142 ^+++^ RG vs. CG: *p* = 0.012 *, r = 0.546 ^+++^ DG vs. CG: *p* = 0.006 **, r = 0.597 ^+++^
Combined multiple techniques		
RG: Pre—Post—Ret	χ^2^(2) = 18.582, *p* < 0.001 *** (Pre: 1.07; Post: 2.54; Ret: 2.39)	Pre vs. Post: *p* < 0.001 ***, r = 0.391 ^++^ Pre vs. Ret: *p* = 0.001 **, r = 0.353 ^++^
DG: Pre—Post—Ret	χ^2^(2) = 24.571, *p* < 0.001 *** (Pre: 1.00; Post: 2.14; Ret: 2.86)	Pre vs. Post: *p* = 0.007 **, r = 0.305 ^++^ Pre vs. Ret: *p* < 0.001 ***, r = 0.496 ^++^
CG: Pre—Post—Ret	χ^2^(2) = 11.259, *p* = 0.004 * (Pre: 1.57; Post: 2.71; Ret: 1.71)	Pre vs. Post: *p* = 0.007 **, r = 0.305 ^++^ Post vs. Ret: *p* = 0.024 *, r = 0.267 ^+^
Pre: RG—DG—CG	χ^2^(2) = 0.288, *p* = 0.866 (RG: 16.39; DG: 17.86; CG: 30.25)	–
Post: RG—DG—CG	χ^2^(2) = 28.127, *p* < 0.001 *** (RG: 14.39; DG: 35.43; CG: 14.68)	RG vs. DG: *p* < 0.001 ***, r = 0.775 ^+++^ DG vs. CG: *p* < 0.001 ***, r = 0.938 ^+++^
Ret: RG—DG—CG	χ^2^(2) = 30.205, *p* < 0.001 *** (RG: 8.21; DG: 34.85; CG: 20.93)	RG vs. DG: *p* = 0.001 ***, r = 0.700 ^+++^ DG vs. CG: *p* < 0.001 ***, r = 1.015 ^+++^

Note. All *p*-values of the post hoc tests are Bonferroni-corrected. RG = repetitive learning group; DG = differential learning group; CG = control group; Pre = pretest; Post = posttest; Ret = retention test. * *p* ≤ 0.05. ** *p* ≤ 0.01. *** *p* ≤ 0.001. ^+^ 0.1 ≤ r < 0.3. ^++^ 0.3 ≤ r < 0.5. ^+++^ r ≥ 0.5.

## Data Availability

The data that support the findings of this study are available from the corresponding author, J.B., upon reasonable request.

## Appendix A

**Table A1:** Training exercises for the DG for the techniques underhand pass, overhand pass, and overhand service divided into different categories.

	**Standing Position**
1	Stand with one leg forward, change leg position while performing.
2	Stand on one leg, change leg while executing.
3	Both legs parallel.
4	Both knees bent, but parallel.
5	Bend knees while performing.
6	Both knees bent, but one leg in front, change leg position while executing.
7	Legs slightly apart.
8	Spread legs slightly while performing.
9	Extend one leg forward.
10	Leg raised, knee bent to chest height, switch with other leg.
11	As in Task 10, but change angle of legs.
12	Stand on the balls of the feet.
13	Stand on the heels of the feet.
	**Trunk Movement**
14	Forward movement during the execution.
15	Sideways movement during execution.
16	Backward movement during execution.
17	Rounding in during execution.
18	Straighten during the execution.
	**Head Movement**
19	Look up.
20	Look down.
21	Head circling.
22	One eye closed.
23	Both eyes blinking.
	**Hand/Arm Movement**
24	Arms higher.
25	Arms (more) forward.
26	Arms sideways.
27	Elbow position slightly back to the side.
28	Elbow position slightly forward and inward.
29	Elbows bent.
30	Elbows extended.
31	Arms crossed.
32	Hands on top of each other.
33	Hands parallel
34	Hands supinated.
35	Hands pronated.
36	Hands as fists.
37	Hands wide open.
	**Mass Movement (Velocity Change)**
38	One step forward during the execution.
39	Two steps forward during the execution.
40	One step backward during execution.
41	Two steps backward during the execution.
42	Side steps to the left and right during execution.
43	Moving one leg forward, backward, left, right during the execution.
44	During execution one quick step forward.
45	During execution one quick step backward.
46	During the execution two quick steps to the front.
47	During execution two quick steps backward.
48	During the execution quick lateral steps to the left and right.
49	Quick one-legged movement forward, backward, left, right during execution.
	**Jumps/Hops**
50	During the execution single-leg hops to the front, back, left, right.
51	While performing two-legged hop forward, backward, left, right.
52	Jump with execution of the technique before landing.
53	Jump with execution of the technique exactly at the landing.
	**Running**
54	Fast runs to the execution position.
55	Fast and slow runs during the performance.
	**Twist and Turns**
56	Half turn left/right on command immediately before execution.
57	Execution with hip position 60°, 120°, 180°, 240°, 300° to the stroke direction.
	**Position Changes**
58	Execution in seated position with legs crossed, legs forward, hurdle seat position, legs wide apart, one leg bent.
59	Execution in a seated position with legs crossed, legs forward, hurdle seat position, legs wide apart, one leg bent.
60	Execution lying bent, stretched, on the side.
61	60, but faster.
62	60, but slower.
63	Execution backwards.
	**Combine at Least two of all Above**
64	Jump and turn left while performing.
65	27 and 46 while execution
66	21 and 32 while execution
67	Different type of balls.
68	Different terrains (e.g., on sand, grass, soft floor mat, …)
